# Quantitative assessment of finger motor performance: Normative data

**DOI:** 10.1371/journal.pone.0186524

**Published:** 2017-10-18

**Authors:** Alessio Signori, Maria Pia Sormani, Irene Schiavetti, Ambra Bisio, Marco Bove, Laura Bonzano

**Affiliations:** 1 Department of Health Sciences, Biostatistics Unit, University of Genoa, Via Pastore,1, Genoa, Italy; 2 Department of Experimental Medicine, Section of Human Physiology, University of Genoa, Viale Benedetto XV, Genoa, Italy; 3 Department of Neuroscience, Rehabilitation, Ophthalmology, Genetics, Maternal and Child Health, University of Genoa, Largo Daneo 3, Genoa, Italy; Universitair Medisch Centrum Groningen, NETHERLANDS

## Abstract

**Background:**

Finger opposition movements are the basis of many daily living activities and are essential in general for manipulating objects; an engineered glove quantitatively assessing motor performance during sequences of finger opposition movements has been shown to be useful to provide reliable measures of finger motor impairment, even subtle, in subjects affected by neurological diseases. However, the obtained behavioral parameters lack published reference values.

**Objective:**

To determine mean values for different motor behavioral parameters describing the strategy adopted by healthy people in performing repeated sequences of finger opposition movements, examining associations with gender and age.

**Methods:**

Normative values for finger motor performance parameters were obtained on a sample of 255 healthy volunteers executing sequences of finger-to-thumb opposition movements, stratified by gender and over a wide range of ages. Touch duration, inter-tapping interval, movement rate, correct sequences (%), movements in advance compared with a metronome (%) and inter-hand interval were assessed.

**Results:**

Increasing age resulted in decreased movement speed, advance movements with respect to a cue, correctness of sequences, and bimanual coordination.

No significant performance differences were found between male and female subjects except for the duration of the finger touch, the interval between two successive touches and their ratio.

**Conclusions:**

We report age- and gender-specific normal mean values and ranges for different parameters objectively describing the performance of finger opposition movement sequences, which may serve as useful references for clinicians to identify possible deficits in subjects affected by diseases altering fine hand motor skills.

## Introduction

Opposable thumbs constitute a crucial feature characterizing fine hand movements in humans [1, 2]; the ability to oppose the fingertip of the thumb to each fingertip of the same hand is the basis of grasping objects of various sizes and operating tools, thus resulting essential in daily living activities (e.g., using a fork, tying shoes, pulling up a zipper, writing). Healthy subjects are able to generate and maintain self-paced rhythmic movement sequences and to synchronize them with external cues [3, 4], engaging different neural pathways. Also, bimanual coordination, which is the ability to use both hands at the same time in a controlled and organized manner, is an important component of motor hand function and is possible since both sides of the brain communicate and share information with each other [5, 6].

Recent studies based on quantitative assessment of finger opposition movements in patients with neurological diseases showed performance impairments with respect to healthy controls [1–6]. In addition, a measure of fine hand motor function has shown to be fundamental when investigating the effects of a motor rehabilitation protocol aiming at improving or maintaining fine movements and coordination skills, allowing comparisons between sessions and groups [7].

In these studies, motor performance during sequences of finger opposition movements was measured by the Glove Analyzer System (GAS, ETT S.p.A., Italy), which is based on a comfortable glove able to record the kinematics of finger-to-thumb opposition movements in unimanual or bimanual conditions. As described in detail previously, a software package records the finger touches with the thumb and provides semi-automatic analysis tools for calculating both spatial and temporal parameters of motor sequences [8]. Furthermore, a magnetic resonance-compatible prototype was developed to analyze finger opposition sequences of different complexities in fMRI environment, thus allowing to investigate the relationships between brain activity during finger opposition movements and the kinematic parameters quantifying finger motor performance acquired simultaneously [9–12].

Importantly, in one study [13] test-retest reliability of the described system has been assessed on a group of healthy subjects performing the same tests one month apart, in order to demonstrate the reproducibility of the finger motor parameters. Then, a large cohort of subjects with multiple sclerosis (MS) was compared to a group of healthy subjects, showing significantly worse performance of repetitive finger opposition sequences with their dominant hand at spontaneous and maximal velocity, and uni- and bi-manually metronome-paced. Finger motor impairment was associated with disease severity, and this methodology was able to discriminate healthy controls and subjects with MS, even with very low disability.

These findings suggest that the proposed tool is able to give reliable measures of finger motor performance that are clinically useful. Indeed, on these bases, there is increasing interest in utilizing the described method in clinical environment and stratified normative ranges need to be implemented to provide viable clinical judgments. A group of healthy volunteers was used in several studies as control group to assess significant differences with respect to the studied patient populations [13–18]; however, up to now normative data on finger motor performance in healthy subjects are lacking.

Therefore, aim of this work was to define age- and gender-specific normal ranges of finger motor performance parameters in a large sample of healthy subjects to build a database of normative data which could be useful for future studies on different populations showing specific alterations of finger opposition movements.

## Materials and methods

### Subjects

Inclusion criteria were: no history or clinical evidence of neurological or psychiatric disorders or use of psychoactive drugs. A total of 255 Italian healthy adults (129 (50.6%) females) with a mean age of 41.3 years (SD: 16 years; range: 20–82 years) were recruited on voluntary basis. All the subjects were right-handed according to a modified Italian-translated Edinburgh Handedness Inventory [19]. The research was carried out according to The Code of Ethics of the World Medical Association (Declaration of Helsinki); the study was approved by our institutional review board and written informed consent was obtained by participating subjects.

### Data collection

**Fig 1:**
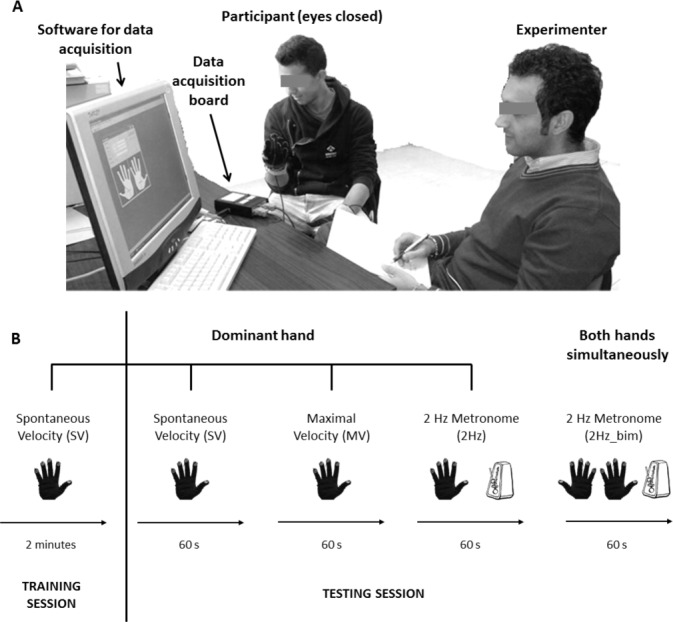
**Typical setup for the execution of the hand motor sequences (Panel A) and timeline of hand motor sequences performed by the subjects (Panel B)**.

Subjects were required to perform finger opposition movements, wearing a sensor-engineered glove on both hands to measure their performance. The experimental set-up is displayed in **Fig 1A**.

In details, five conducting metal sensors are located on the palmar surface of the distal phalanxes of the glove, like fingerprints, in order to record the contact during opposition movements between the thumb and another finger (detecting a square wave corresponding to contact/no contact) [8, 10]. Data were acquired at 1 KHz by means of a data acquisition board (USB-1208FS, Measurement Computing, USA). An ad hoc software tool generated the acoustic pacing signal, which was delivered by the system and listened by the subjects through isolation headphones, and recorded the occurrence of each tone and of each finger touch in the motor sequence. As previously reported, the program used to provide the cue and for data recording and analysis was developed with the Microsoft Visual Studio 2013,.NET Framework 4.5, written in C# exploiting the Windows Presentation Foundation for desktop application development platform [10].

### Experimental protocol

The experimental protocol consisted of a simple sequence of finger opposition movements (thumb-to-index, medium, ring and little finger) repeated continuously for 60 s, with the dominant (right) hand. This task had to be performed in different experimental conditions, as illustrated in **Fig 1B**: at spontaneous (SV condition) and maximal (MV condition) velocity, and paced with a metronome tone set at a rate of 2Hz (2Hz condition), i.e., about the spontaneous speed of healthy subjects [8]. The 2Hz condition was also performed with both hands simultaneously (2Hz_bim condition) to assess bimanual coordination. An eyes-closed paradigm was chosen to allow the participant to focus on the motor task and on the auditory cue (in the 2Hz conditions), without being distracted from external and non-pertinent visual cues. The testing session included four 60-s trials (one per condition); the order of the trials was randomly assigned to the subjects, and the corresponding instructions were given to the subjects before starting. A familiarization phase preceded the actual recording, in which all subjects practiced the task at their own spontaneous pace until they were able to perform the finger motor sequence without errors; training ended generally within 2 min. It should be noted that the evaluation protocol was not based on a learning process, considering that: (i) the proposed finger motor sequence was rather simple because there was no irregular order of finger touches to learn; (ii) the finger motor parameters were averaged over the entire recording period of 60 s and not over several trials, conversely to what occurs in learning paradigms; (iii) test-retest reliability of the adopted protocol was previously assessed, showing the reproducibility of the finger motor parameters [13].

### Data analysis

Different parameters were registered for the specific analysis, as previously described in [13]: Touch Duration (TD), calculated as the contact time between the thumb and another finger; Inter Tapping Interval (ITI), defined as the time interval between the end of a contact and the beginning of the subsequent contact in the finger motor sequence; movement rate (RATE) computed as 1/(TD+ITI).

Movement rate was considered the outcome parameter in the condition of spontaneous and maximal velocity, whereas TD, ITI and TD/ITI were chosen to describe the performance when finger opposition movements were paced with a metronome (2Hz). In the latter condition, to assess temporal accuracy we calculated the percentage of touches preceding the metronome tone over the total number of correct touches (percentage of advance movements—%ADV_MOV) [9, 11]. In addition, to assess spatial accuracy we calculated the percentage of sequences performed correctly over the total number of sequences (percentage of correct sequences—%CORR_SEQ); in particular, we investigated the spatial accuracy related to the finger touched (and not to how precisely it was touched), which could result wrong or right with respect to the predefined sequence of finger touches. For the bimanual trial, Inter Hand Interval (IHI) was calculated as the absolute time difference between the touch onset occurring in the left hand and the corresponding touch in the right hand; according to this definition, larger values indicate reduced ability in bimanual coordination [16].

### Statistical analysis

Mean values with standard deviation (SD) were reported for all the analyzed finger motor performance parameters. Mean age of males and females was compared by means of independent samples t-test. Pearson’s correlation coefficient between age of subjects and all parameters was calculated. A general linear model with Gaussian distribution was used to predict the mean performance and define the age-dependent normal ranges for RATE, TD, ITI, TD/ITI and IHI. A log-transformation was applied to IHI to adjust for its skewed distribution. Different polynomials of age (age^2^,age^3^, age^1/2^, age^1/3^) were tested to define the model better fitting our data.

Normal ranges were calculated as the mean predicted value ± 1.96* SD of residuals obtained by the model with the best fitting [20–22].

For %CORR_SEQ and %ADV_MOV a general linear model with binomial distribution and logit link function was adopted to predict the mean performance and define the age-dependent normal ranges. Since the residuals had a skewed distribution, to determine the normal ranges for these two parameters the 2.5 and 97.5 percentiles, instead of SD, of residuals were used.

For each motor parameter, differences between males and females and the interaction between gender and age (age*gender) were assessed including these characteristics into the general linear model.

For all models the robust standard errors were used. This permitted to be robust to some kind of misspecification. A p-value lower of 0.05 was considered statistically significant. Stata (v.14; StataCorp.) was used for computation.

## Results

The demographic characteristics of the enrolled subjects and the protocols performed are reported in **Table 1**.

**Table 1:** Demographic characteristics and experimental protocols performed by the included subjects with the GAS system.

**Demographic characteristics**	**N = 255**
Age, mean ± SD (min-max)	41.3±16.0 (20–80)
Females	129 (50.6)
Males	126 (49.4)
**Protocols**	**N (%)**
Spontaneous Velocity (SV)	245 (96.1)
Maximal Velocity (MV)	245 (96.1)
Metronome condition (2Hz)	249 (97.6)
Bimanual metronome condition (2Hz_bim)	216 (84.7)

SD: standard deviation; SV = Spontaneous Velocity condition. MV = Maximal Velocity condition. 2 Hz = metronome condition (tone set at a rate of 2 Hz). 2 Hz_bim = metronome condition with both hands simultaneously.

No significant age differences (p = 0.36) were observed between males (42 ± 16.1 years, range: 20–82 years) and females (40.6 ± 16 years, range: 20–79 years). **Table 2** shows the finger motor performance parameters with their range (minimum-maximum); **Table 3** shows the association of the same parameters with age and gender.

**Table 2:** Finger motor performance measured by means of the GAS system.

Parameter	Protocol	Mean±SD	Range (min-max)
RATE [Hz]	SV	2.21±0.49	1.06–4.08
	MV	3.03±0.61	1.13–5.91
TD [ms]	2 Hz	209.9±48.9	79.9–380.7
ITI [ms]	2 Hz	289.1±55.2	116.9–477
TD/ITI	2 Hz	0.87±0.43	0.19–3.76
% CORR_SEQ	2 Hz	79.7±20.2	8.3–100
%ADV_MOV	2 Hz	76.3±25	0–100
IHI (log-transformed)	2 Hz_bim	3.17±0.66	1.94–5.58

SD: standard deviation; SV = Spontaneous Velocity condition. MV = Maximal Velocity condition. 2 Hz = metronome condition (tone set at a rate of 2 Hz). 2 Hz_bim = metronome condition with both hands simultaneously. RATE = movement speed. TD = Touch Duration. ITI = Inter Tapping Interval. % CORR_SEQ = sequences correctly performed. %ADV_MOV = percentage of touches preceding the metronome tone. IHI = Inter Hand Interval.

**Table 3:** Relationship of finger motor performance parameters with age and gender.

Parameter	Protocol	Age		Gender*			Interaction age*gender (p-value)
		r	p-value	Females	Males	p-value	
RATE [Hz]	SV	-0.18	0.013	2.19 ± 0.47	2.23 ± 0.50	0.40	0.61
	MV	-0.32	<0.001	2.98 ± 0.59	3.09 ± 0.62	0.19	0.54
TD [ms]	2Hz	0.05	0.40	216.1 ± 51.3	203.6 ± 45.8	0.045	0.30
ITI [ms]	2Hz	0.02	0.75	282 ± 55.4	296.3 ± 54.3	0.041	0.22
TD/ITI	2Hz	0.13	0.049	0.94 ± 0.50	0.80 ± 0.34	0.008	0.50
% CORR_SEQ	2Hz	-0.18	0.005	80.6 ±19.9	78.8 ± 20.6	0.48	0.75
%ADV_MOV	2Hz	-0.25	<0.001	78.9 ± 23.3	73.6 ± 26.4	0.098	0.01
IHI [ms] (log-transformed)	2Hz_Bim	0.41	<0.001	3.19 ± 0.63	3.15 ± 0.68	0.49	0.039

*Results reported as mean±standard deviation

IQR: inter-quartile range; SV = Spontaneous Velocity condition. MV = Maximal Velocity condition. 2 Hz = metronome condition (tone set at a rate of 2 Hz). 2 Hz_bim = metronome condition with both hands simultaneously. RATE = movement speed. TD = Touch Duration. ITI = Inter Tapping Interval. %CORR_SEQ = sequences correctly performed. %ADV_MOV = percentage of touches preceding the metronome tone IHI = Inter Hand Interval.

### Rate

When performing the task at spontaneous velocity (SV) or at maximal velocity (MV) a significant decrease (**Table 3**) of movement rate with increasing age was observed (SV: r = -0.18, p = 0.013; MV: r = -0.32; p<0.001). In both conditions no significant effect of gender (SV: p = 0.40; MV: p = 0.19) and no significant interactions between age and gender (SV: p = 0.61; MV: p = 0.54) were found.

**Fig 2:**
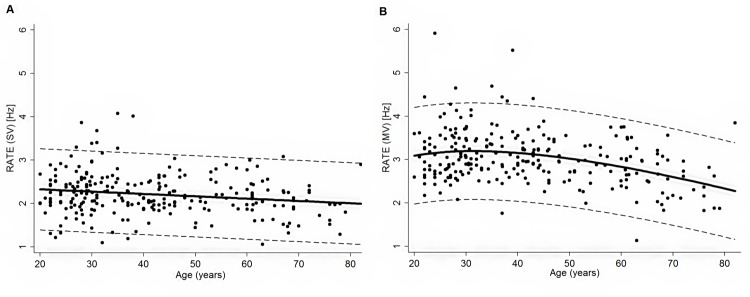
**Scatter plot of the individual values of RATE in the SV condition (Panel A) and MV condition (Panel B).** Data from the male and female subjects are pooled together; solid lines represent the mean predicted value while dashed lines represent the normal ranges.

**Fig 2** shows the distribution of movement rate according to age, together with the predicted trajectory and the estimated normal intervals.

The best trajectory was linear for the SV condition whilst it was represented by the sum of linear and cube root component of age for the MV condition. In fact, for the MV condition RATE increased up to 35 years of age followed by a decrease in older ages. Normal ranges according to age and sex can be calculated in the Excel file in **S1 File**.

### Touch duration and Inter Tapping Interval

Higher touch duration and lower inter-tapping interval were found in females as compared to males (TD: p = 0.045; ITI: p = 0.041), whilst no correlation with age (TD: p = 0.40; ITI: p = 0.75) was observed (**Table 3**).

No significant interaction between gender and age was detected (TD: p = 0.30; ITI: p = 0.22).

The estimated normative interval for TD ranged between 115.6 ms and 330.9 ms in females and between 101.6 ms and 297.4 ms in males, while for ITI the two intervals were, respectively, 166.4 ms to 387.6 ms and 200.6 ms to 410.9 ms.

A significant increase in the ratio between TD and ITI (TD/ITI) with increasing age was observed (r = 0.13; p = 0.049) together with a trend in higher values for females (p = 0.08; **Table 3; S1 Fig**).

### Percentage of correct sequences

**Fig 3:**
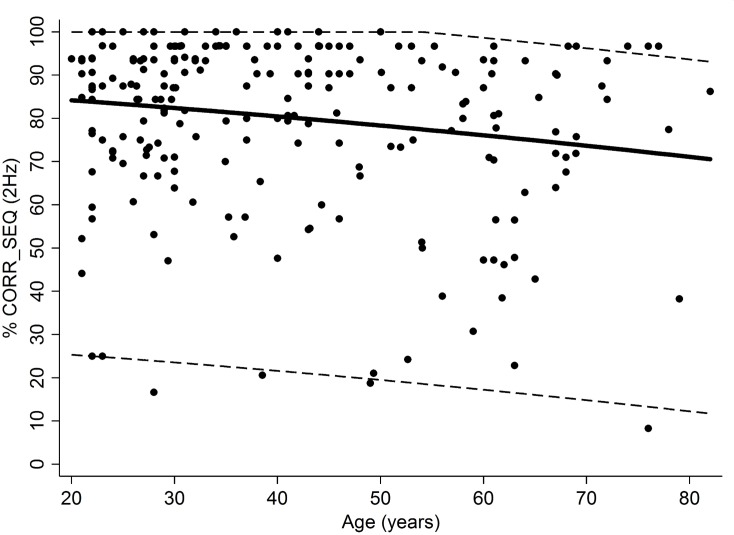
Scatter plot of the individual values of percentage of correct sequences in the 2Hz condition. Data from the male and female subjects are pooled together; solid lines represent the mean predicted value while dashed lines represent the normal ranges.

Older subjects showed a significant decrease in %CORR_SEQ (r = -0.18; p = 0.005), with no differences between males and females (p = 0.48) and no interaction between age and gender (p = 0.75). Reference ranges according to age were predicted and plotted in **Fig 3**.

### Percentage of advance movements

Older subjects showed a decrease in %ADV_MOV (r = -0.25; p<0.001); males showed lower performance than females, but this difference was not statistically significant (p = 0.098). A significant interaction between age and gender (p = 0.01) was found. Normal ranges according to age and stratified for gender are shown in **S2 Fig.**

### Inter hand interval

**Fig 4:**
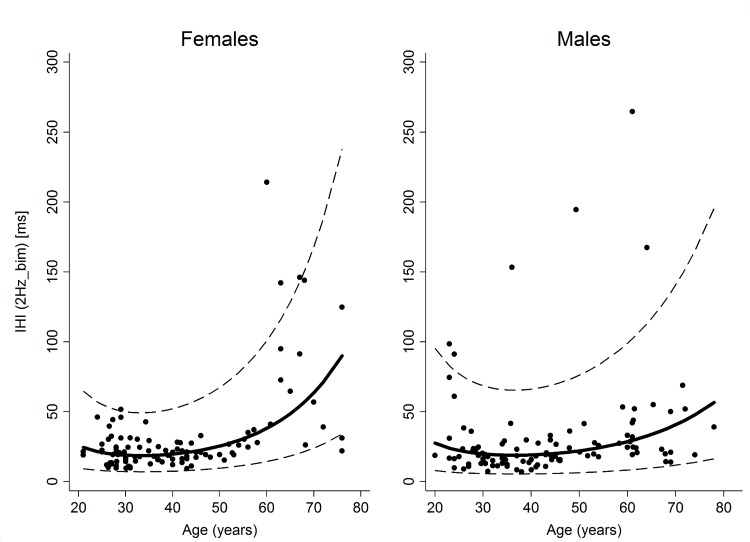
Scatter plot of the individual values of inter hand interval in the 2Hz_bim condition. Solid lines represent the mean predicted value while dashed lines represent the normal ranges.

A significant worsening of IHI with increasing age was detected (r = 0.41; p<0.001). No significant differences between males and females were observed (p = 0.49), while there was a significant interaction between age and gender (p = 0.039). For this reason the normative values were presented by age and gender (**Fig 4**).

## Discussion and conclusions

Aim of this study was to define age- and gender- stratified normative data for finger motor performance obtained by means of an engineered glove [8]. The idea of this work derived from the increasing interest in applying the described methodology in a clinical context to objectively assess impairments during sequences of finger opposition movements in different neurological diseases, such as multiple sclerosis [13, 16–18], Charcot-Marie-Tooth disease [14], and Parkinson’s disease [23]. Having reference intervals from healthy subjects for the different parameters describing finger motor performance represent the basis to interpret the results obtained in patients performing finger opposition movement sequences. In particular, we hypothesized an influence of age on the glove-derived parameter values and possible performance differences between males and females. Therefore, data obtained from a large sample of healthy adults (20–82 years of both sexes) were analyzed with respect to age and gender.

The only significant difference according to gender was observed for TD and ITI and their ratio TD/ITI when right-hand finger movements were metronome-paced at 2Hz. In fact TD was longer and ITI was shorter in female with respect to male subjects. However, these differences have no important implications, indicating for instance a different strategy in performing the sequence of finger touches, given that TD/ITI ratio was lower than 1 in both groups.

On average, RATE decreased and IHI increased with age, indicating that subjects became slower and showed worse coordination between the two hands. These findings are in line with the scientific literature showing that aging is source of motor performance decline due to multi-joint and interlimb coordination difficulties [24, 25], increased variability of movement [26], slowing of movement [27, 28].

In particular, we can suggest that the decline in bimanual coordination is related to reduced microstructural integrity of the corpus callosum [16, 29]. This is supported by studies based on diffusion tensor imaging demonstrating age-related reductions of white matter integrity in association with similar declines in interhemispheric transfer in normal healthy adults, with equivalent trend in men and women and linear from about age 20 years onwards [30]. In another review, age-related declines have been shown in callosal size and integrity and have been considered a key contributor to unimanual and bimanual control deficits [31]. In fact, callosal fiber tracts are essential for inhibiting the ipsilateral motor cortex during both unimanual and bimanual control [32–34].

The corpus callosum is commonly affected in MS [35], in which demyelination causes deficits in the conducting properties of axons, altering electrical signaling in the central nervous system and thus influencing motor performance. Then, the major application of the presented methodology has been in research studies on subjects affected by MS, demonstrating impaired finger motor functions. Importantly, in the context of MS clinical trials, we could suggest to objectively assess the disability impact on fine hand motor functions by means of quantitative measurements of finger opposition movements, integrating current methods for disability assessment which are heavily weighted toward ambulation [13]. This previous work also demonstrated high sensitivity of the presented tool, which could be crucial for monitoring the disease course and the treatment effects starting from the early phase of the disease. In clinical practice, the obtained finger motor parameters could integrate the neurological examination and help a clinician to choose between early pharmacological treatment or tailored rehabilitation treatment. Indeed, this methodology could be able to detect subtle impairment when the neurological examination still revealed no hand impairments, in favor of earlier diagnosis and better disability characterization. Also, it could have a central role in assessing hand disability, allowing a better implementation of a rehabilitation protocol, in the progressive phase of the disease when patients may need to use a wheelchair and finger movements are even more important to daily life and require temporal and spatial coordination. For instance, a detailed analysis of finger motor performance could help balance unimanual and bimanual voluntary exercises in each session along the rehabilitation program, and provide objective information on the treatment effects [7].

Then, the investigation of externally cued motor tasks measured by means of the glove system could provide interesting results in Parkinson's disease, in comparison with internally-paced movements and in relation to subjective fatigue [23]. Furthermore, we could suggest similar applications to other conditions affecting hand function (e.g., stroke, carpal tunnel syndrome, muscular dystrophies). In this framework, another example is one study in which the engineered glove was applied to detect hand dysfunction in a population of subjects affected by Charcot-Marie-Tooth disease, i.e., the most common inherited neuropathy. In particular, this methodology was found to disclose subclinical hand impairment in patients with clinically unaffected hands disease [14].

Lastly, other studies should be required to test the proposed system also on pathological children, since this tool could be easily applied also in pediatric clinical practice. To achieve this goal, normative data should be collected from children with different ages, where the developing central nervous system could strongly influence the recorded performance. Until now, one study was conducted on a pediatric sample, demonstrating no problems in administering the protocol and sensitivity to differentiate between the finger motor performance of subjects with Tourette Syndrome and age-matched healthy controls [36].

In conclusions, we reported mean values for different parameters objectively describing the performance of finger opposition movement sequences in healthy people. These values were obtained by means of an assessment technique, which is based on a simple system and protocol, previously demonstrated to be reproducible across trials and widely acceptable by patients [13]. In particular, here data were collected from a large group of healthy subjects and stratified by age and gender, demonstrating that finger motor parameters worsened with increasing age, whilst there were no substantial differences between males and females.

We think that the applicability of this methodology is wide in the evaluation of patients affected by different pathologies. Indeed, findings from the present study may serve as useful references for clinicians to identify possible deficits in subjects affected by diseases altering fine hand motor skills.

## Supporting information

S1 Fig **Scatter plot of the individual values of TD/ITI ratio in the 2Hz condition stratified for females (Panel A) and males (Panel B).** TD: touch duration; ITI: inter-tapping interval; Solid lines represent the mean predicted value while dashed lines represent the normal ranges.
(TIF)

S2 Fig **Scatter plot of the individual values of % ADV_MOV in the 2Hz condition stratified for females (Panel A) and males (Panel B).** Solid lines represent the mean predicted value while dashed lines represent the normal ranges.
(TIF)

S1 File Excel calculator for normal ranges of hand motor parameters. (XLSX)

S2 File Database with all performances from 255 healthy subjects. (XLS)

## Abbreviations

fMRI: functional Magnetic Resonance Imaging
GAS: Glove Analyzer System
IHI: Inter-hand interval
ITI: Inter tapping interval
MS: Multiple Sclerosis
MV: Maximum velocity
SD: standard deviation
SV: Spontaneous velocity
TD: Touch duration
