# Supplementary figures and images for: Quantitative assessment of finger motor performance: Normative data
Source: PLoS One. 2017 Oct 18;12(10):e0186524. doi: 10.1371/journal.pone.0186524 (PMC5646860; doi:10.1371/journal.pone.0186524)

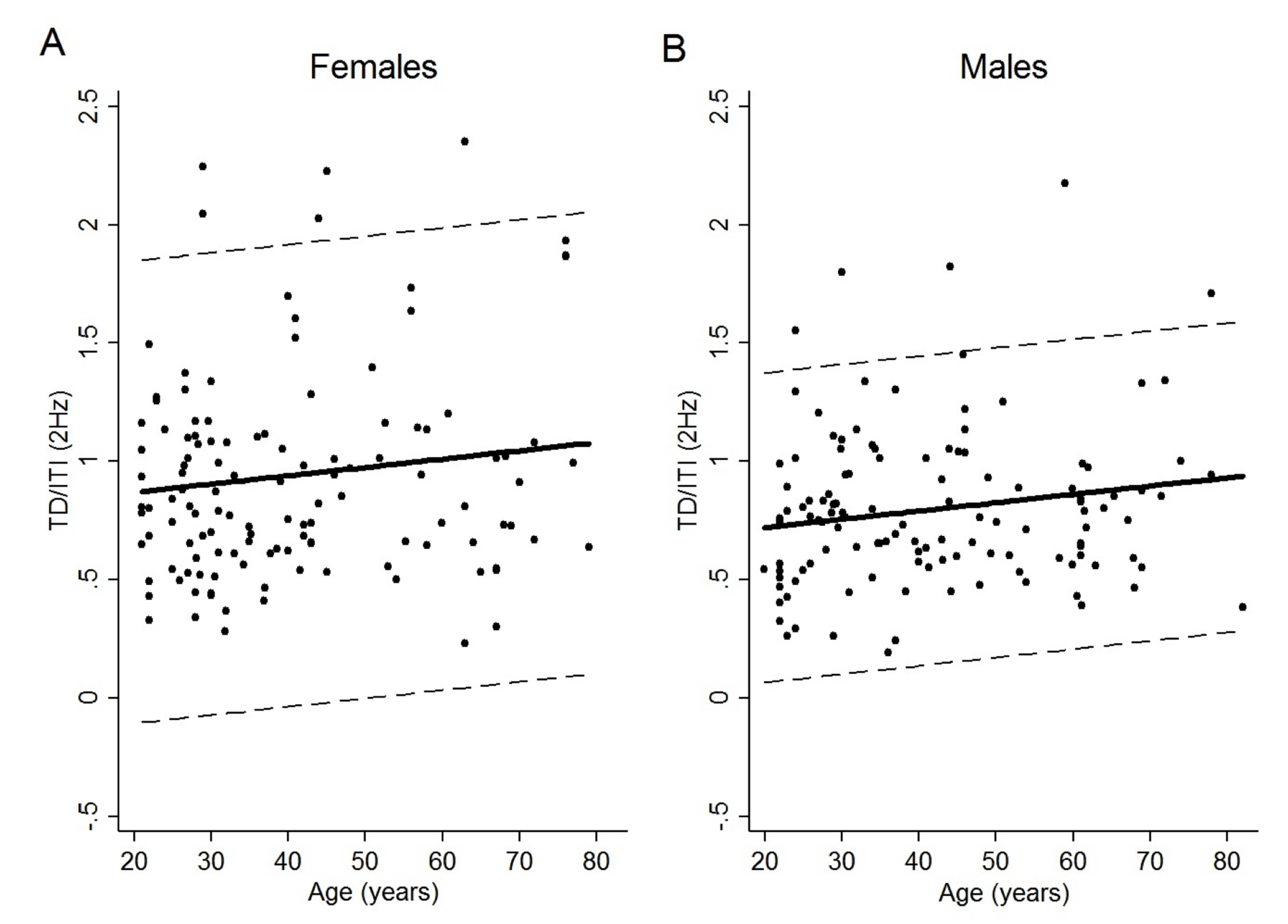

Supplement: S1 Fig — Scatter plot of the individual values of TD/ITI ratio in the 2Hz condition stratified for females (Panel A) and males (Panel B). TD: touch duration; ITI: inter-tapping interval; Solid lines represent the mean predicted value while dashed lines represent the normal ranges. (TIF) [file pone.0186524.s001.tif]

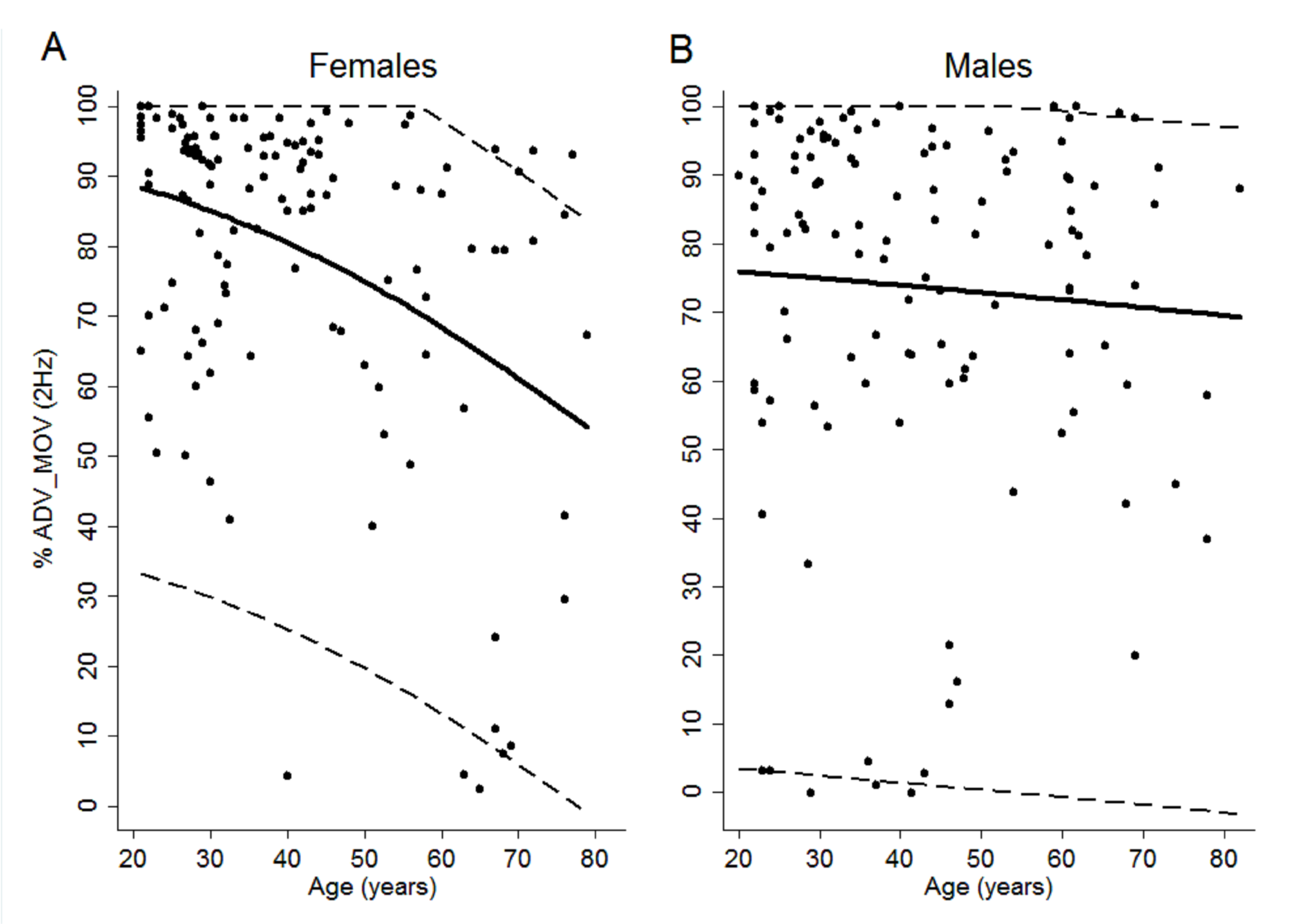

Supplement: S2 Fig — Scatter plot of the individual values of % ADV_MOV in the 2Hz condition stratified for females (Panel A) and males (Panel B). Solid lines represent the mean predicted value while dashed lines represent the normal ranges. (TIF) [file pone.0186524.s002.tif]
